# Drainage in Surgical Treatment

**Published:** 1894-01-20

**Authors:** W. Horrocks

**Affiliations:** Surgeon to the Bradford Infirmary


### DRAINAGE IN SURGICAL TREATMENT.

By W. Horrocks, M.B., F.R.C.S., Surgeon to the Bradford Infirmary.

Drainage has become so much a part of surgical treatment that there is danger of using it in a routine way without considering its advantages or drawbacks. The object of drainage is to allow the escape of fluid which would accumulate and separate the wound surfaces. This fluid in recent aseptic wounds is blood which oozes from the capillaries, and serum which is caused to exude by mechanical injuries of the operation and chemical irritation by antiseptic lotions.

In wounds where suppuration has occurred drainage has additional advantages. In operations on bone the bone wound or cavity has unyielding walls, which do not fall together. The cavity is soon filled with blood-clot. If the wound is aseptic, this forms an excellent scaffolding for the formation of new bone. If, however, any pus-producing cells remain, the mass of clot soon breaks down into pus; hence, in septic or doubtful bone wounds it is desirable to drain. In the treatment of empyema the walls of the abscess, *i.e.*, the opposing pleural surfaces, heal by granulation. This necessitates prolonged drainage.

There are certain essentials in drainage of wounds. 1. The escape opening should have a dependent position, so that the flow of fluid is aided by gravitation. An exception occurs in peritoneal drainage through an opening above the pubes. Fluid in the abdomen is under positive pressure from the elastic abdominal walls and the gas-distended intestines. That intra-abdominal pressure is sometimes considerable is seen by the forced expulsion of ascitic fluid through a puncture of the abdominal wall. The rise of fluid in the glass drainage-tube is due to intra-abdominal pressure overcoming gravity. Capillary attraction, which is used in some methods of drainage, may act through a raised outlet.

2. A free outlet from the drain is essential. This may be hindered during the first days of healing by blockage of the tube by clot. The clot may be removed by suction with a pipette, not by forcible injection with a syringe. Later the tube may become blocked by the growth of granulations through its opens. To obviate this, the tube should be rotated each time the wound is dressed.

3. The inner end of the drain should reach the lowest part of the cavity. This is especially necessary in peritoneal drainage. The end of the tube must reach the lowest part of "Douglas" pouch, or the cavity outside each kidney, while its extremity should be free from blockage by intestine pressed against its open end.

Ideal wound treatment teaches the removal of all foreign bodies, therefore the use of a drainage tube is only allowable if it is shown to prevent a greater evil. In large aseptic wounds considerable oozing of blood and exudation of serum takes place after the wound is closed. The question to decide is, whether it is advisable to leave the fluid to be absorbed by the lymphatics, or to allow it to escape by a drainage tube. Various methods of drainage have been tried. The following are most commonly used:—

1. *Rubber Tubing.*—Indiarubber tubing made with Para rubber of various sizes, with holes cut at intervals to allow the fluid to find its way into the tube. The outer end of the tube should be cut level with the skin surface, and secured with a safety pin. In aseptic wounds the drain is left in the wound for two days. It is then removed, and the lips of the opening brought together with a suture inserted at the time of the operation.

2. *Glass Drainage Tube.*—This is chiefly used for drainage of the peritoneal cavity. After the operation a pipette is passed to the lowest part of the drain, which is cleared of clot by suction. This suction is repeated every two hours during the first day. On the following days the serum is removed until it becomes clear and small in amount. A small piece of rubber tube is then passed inside the glass tube to its lower end, and the glass tube is withdrawn. The rubber tube is left in for two or three days, then removed.

3. *Tampons.*—Tampons are made of strips of iodoform, or sublimated gauze, and are used to pack cavities, from whose walls there will probably be considerable oozing. In excision of the hip or ankle, deep, silkworm gut sutures are passed through the lips of the wound and left loose. The deep parts of the wound are packed with strips of gauze, whose ends are left out. On the second day the tampons are removed and the sutures tightened.

4. *Horse-Hair.*—Strands of horse-hair make a useful drain in small wounds. It acts by capillary attraction along the spaces between the hairs. Catgut in strands has been used for this purpose, but is unsuitable. The catgut swells during the first twenty-four hours, and the fibres become closely pressed against each other. This obliterates the capillary spaces, and converts the drain into a plug.

